# A comparative study of intestinal *Pseudomonas aeruginosa* in healthy individuals and ICU inpatients

**DOI:** 10.1186/s44280-023-00014-y

**Published:** 2023-05-30

**Authors:** Yanyan Hu, Siheng Wang, Yanyan Zhang, Yuchen Wu, Congcong Liu, Xiaoyang Ju, Hongwei Zhou, Chang Cai, Rong Zhang

**Affiliations:** 1 Department of Clinical Laboratory, School of Medicine, Second Affiliated Hospital of Zhejiang University, Hangzhou, 310009 China; 2 Australia Joint Laboratory for Animal Health Big Data Analytics, College of Animal Science and Technology, Zhejiang Agricultural and Forestry University, Hangzhou, China

**Keywords:** Intestinal *Pseudomonas aeruginosa*, Intensive care unit, Healthy individuals, Virulence, *Klebsiella pneumoniae* carbapenemase

## Abstract

The human intestinal tract is considered the most important reservoir of the opportunistic pathogens, including *Pseudomonas aeruginosa, *which is often overlooked but critical due to its antimicrobial resistance and virulence. Public health interventions to control this pathogen require a comprehensive understanding of its epidemiology and genomics.  In the current study, we identified *P. aeruginosa* strains from 2,605 fecal samples collected between 2021 to 2022. Among these samples, 574 were from ICU inpatients in Zhejiang province, while 2,031 were obtained from healthy individuals residing in ten different provinces in China. The prevalence of *P. aeruginosa* intestinal carriage was found to be higher in ICU inpatients (10.28%, 95% CI: 7.79%–12.76%) than that in healthy individuals (3.99%, 81/2,031, 95% CI: 3.14%–4.84%). Similarly, the prevalence of carbapenem-resistant *P. aeruginosa* (CRPA) was higher in ICU inpatients (32.2%) compared to healthy individuals (7.41%). The population structure analysis of our isolates revealed a predominantly non-clonal distribution, with 41 distinct sequence types identified among 59 *P. aeruginosa* isolates from ICU inpatients and 38 different STs among 81 *P. aeruginosa* isolates from healthy individuals. These findings suggest that the individual acquisition of *P. aeruginosa* is more frequent than patient-to-patient transmission, as evidenced by the polyclonal population structure. Antimicrobial susceptibility testing and genome analysis indicated that *P. aeruginosa* strains from ICU inpatients exhibited significantly higher resistance rates to most antimicrobials and harbored a greater number of acquired resistance genes compared to strains from healthy individuals. Notably, in ICU inpatients, we identified three isolates of ST463, all of which shared the conserved Tn*3*-TnpR-IS*Kpn8*-*bla*_KPC_-IS*Kpn6* genetic context. Additionally, five isolates carrying the *qac*E gene were also identified, these findings suggest that small-scale transmission events may still occur within the ICU setting, posing significant challenges for clinical management. With regard to virulence factors, we observed similar profiles between the two groups, except for *phzA2*, *phzB2,* and *pilA*, which were statistically higher in isolates from healthy individuals. This may be because the accumulating resistance mutations in ICU-derived *P. aeruginosa* are linked to a decrease in virulence.

## Introduction

*Pseudomonas aeruginosa* is found in a diverse range of environments due to its ability to tolerate various growth conditions [1]. As a major opportunistic pathogen, it is frequently associated with hospital-acquired infections, particularly in burn units and intensive care units (ICUs). The World Health Organization and the United States Centers for Disease Control and Prevention have both classified *P. aeruginosa* as one of the top critical pathogens in healthcare settings due to the emergence of clinical multidrug-resistant isolates [2, 3].

One of the major challenges with clinical *P. aeruginosa* isolates is antimicrobial resistance and virulence*. P. aeruginosa* exhibited high-level resistance profiles due to its intrinsic resistance to most antimicrobial agents [4]. Carbapenems are considered the most effective antimicrobial agents against severe nosocomial infections caused by *P. aeruginosa* [5]. However, the emerging and high prevalence of carbapenem-resistant *P. aeruginosa* (CRPA) has already posed a significant threat to public health worldwide [2]. Moreover, various virulence factors such as type III effectors, type VI effectors, and adherence factors have been demonstrated to contribute to *P. aeruginosa* infection [6] and also play crucial roles in the high mortality in clinical settings [7].

The human intestinal tract is considered one of the most important reservoirs of *P. aeruginosa.* Gut barrier translocation of endogenous intestinal *P. aeruginosa* is a significant pathogenic phenomenon that can cause systemic infections, particularly in immunocompromised patients [8]. *P. aeruginosa* can cause pulmonary infection through either the oral-fecal route of transmission or spread from the intestine to the lungs via the bloodstream [9]. Patients in ICU who are colonized with *P. aeruginosa* in their intestines have a significantly higher mortality rate than those without such colonization [10]. Additionally, a previous study has also reported that CRPA can reach 30% in the intestinal *P. aeruginosa* [11]. At present, however, there is no systematic comparison of the prevalence, antimicrobial resistance, and virulence of intestinal *P. aeruginosa* strains between healthy individuals and ICU inpatients. Therefore, the aim of our study is to investigate the features of intestinal colonization of *P. aeruginosa* in these two groups in order to provide a better understanding of this pathogen.

## Results

### Prevalence of intestinal *P. aeruginosa* in ICU inpatients and healthy individuals

**Fig. 1:**
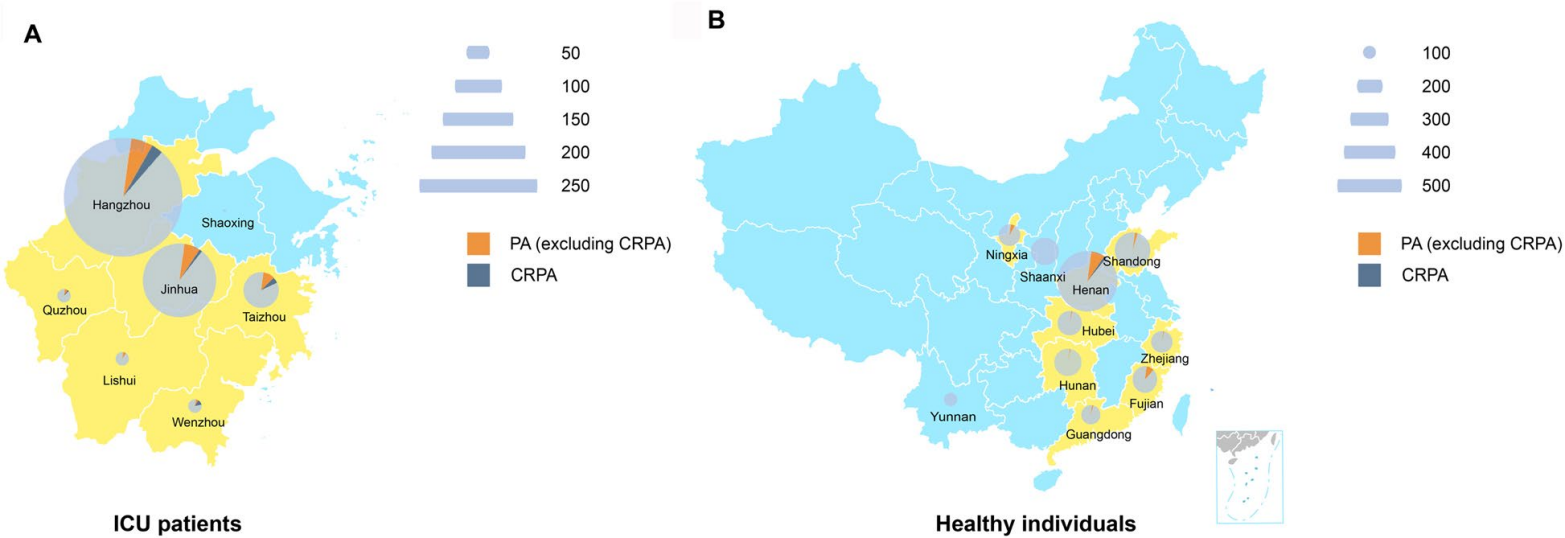
The detailed strain distribution of intestinal *P. aeruginosa* strains. **A** Strains collected from ICU inpatients in Zhejiang Province. **B** Strains obtained from healthy individuals in China

A total of 574 fecal samples were obtained from ICU inpatients, from which 59 (10.28%, 95% CI: 7.79%–12.76%) *P. aeruginosa* strains were identified, with 33 (9.62%, 95% CI: 6.50%–12.74%) strains from 343 samples in 2021 and 26 (11.26%, 95% CI: 7.18%–15.33%) from 231 samples in 2022. A total of 2,031 fecal samples were gathered from healthy individuals in ten Chinese provinces, from which 81 (3.99%, 95% CI: 3.14%–4.84%) *P. aeruginosa* strains were isolated in eight provinces (Fig. 1), with 29 (2.89%, 95% CI: 1.85%–3.92%) strains from 1,004 samples in 2021 and 52 (5.06%, 95% CI: 3.72%–6.40%) from 1,027 samples in 2022. Overall, ICU inpatients had considerably greater intestinal *P. aeruginosa* carriage than healthy individuals (Table 1). In addition, 32.20% (19/59, 95% CI: 20.28%–44.13%) ICU-derived strains (*n* = 19) belonged to CRPA, which was significantly higher than 7.41% (6/81, 95% CI: 1.70%–13.11%) strains (*n* = 6) from healthy individuals identified to be CRPA (*P* < 0.001). Nineteen strains (32.20%, 19/59, 95% CI: 20.28%–44.13%) of *P. aeruginosa* from ICU inpatients belonged to the CRPA. Only six strains (7.41%, 6/81, 95% CI: 1.70%–13.11%) of *P. aeruginosa* from healthy individuals were identified to be CRPA, which was significantly lower than the proportion in the samples of ICU inpatients (*P* < 0.001).

**Table 1:** Prevalence of *P. aeruginosa* from ICU inpatients and healthy individuals

Year	Isolation rate of *P. aeruginosa*		*P* value	Isolation rate of CRPA^a^		*P* value
	ICU inpatients (%) (95% CI^b^)	healthy individuals (%) (95% CI^b^)		ICU inpatients (%) (95% CI^b^)	healthy individuals (%) (95% CI^b^)	
2021	9.62 (6.50–12.74)	2.89 (1.85–3.92)	** < 0.001**	39.39 (22.72–56.07)	0.00 (0.00–0.00)	** < 0.001**
2022	11.26 (7.18–15.33)	5.06 (3.72–6.40)	** < 0.001**	23.08 (6.88–39.27)	11.54 (2.85–20.22)	0.318
Total	10.28 (7.79–12.76)	3.99 (3.14–4.84)	** < 0.001**	32.20 (20.28–44.13)	7.41 (1.70–13.11)	** < 0.001**

^a^The proportion of CRPA in *P. aeruginosa*

^b^CI Confidence interval

### Antimicrobial susceptibility profiles

*P. aeruginosa* isolated from ICU inpatients had significantly higher resistance rates to most antimicrobials than that from healthy individuals (Table 2). None of the commonly used antibacterial agents (such as meropenem, ceftazidime, cefepime, piperacillin/tazobactam, cefoperazone/sulbactam, aztreonam, ceftazidime/avibactam, and gentamicin) resistant strains were isolated in intestinal *P. aeruginosa* from healthy individuals. However, *P. aeruginosa* strains collected from ICU inpatients showed 3.39% ~ 20.34% resistance rates against the above antibiotics (Table 2). In addition, it should be noted that imipenem-resistant (MIC = 8 ug/ml) *P. aeruginosa* (*n* = 6, 7.41%,) was observed in healthy individuals. In addition, levofloxacin had the highest resistance rate among intestinal *P. aeruginosa* isolates in ICU inpatients (30.51%) and healthy individuals (12.35%, Table 2).

**Table 2:** Antimicrobial susceptibility of *P. aeruginosa* from ICU inpatients and healthy individuals

Antimicrobial agent^a^	ICU inpatients				healthy individuals				*P* value ^b^
	MIC_50_ (μg/ml)	MIC_90_ (μg/ml)	R%	S%	MIC_50_ (μg/ml)	MIC_90_ (μg/ml)	R%	S%	
IPM	≤ 2	8	28.81	66.10	≤ 2	4	7.41	80.25	** < 0.001**
MEM	≤ 2	8	20.34	69.49	≤ 2	≤ 2	0.00	95.06	-
CAZ	≤ 2	64	20.34	76.27	≤ 2	≤ 2	0.00	100.00	-
FEP	4	32	11.86	79.66	≤ 2	≤ 2	0.00	100.00	-
AK	2	4	0.00	100.00	2	2	0.00	100.00	-
LEV	≤ 0.5	> 16	30.51	55.93	≤ 0.5	4	12.35	85.19	**0.008**
CIP	≤ 0.25	> 8	27.12	67.80	≤ 0.25	1	1.23	85.19	** < 0.001**
CO	1	1	0.00	100.00	1	1	0.00	100.00	-
TZP	≤ 8	128	15.25	77.97	≤ 8	≤ 8	0.00	98.77	-
SCF	≤ 8	64	13.56	76.27	≤ 8	≤ 8	0.00	100.00	-
ATM	≤ 4	32	11.86	66.10	≤ 4	8	0.00	98.77	-
CAV	2	8	3.39	96.61	1	2	0.00	100.00	-
GM	1	2	5.08	94.92	1	1	0.00	100.00	-

^a^ *IPM* Imipenem, *MEM* Meropenem, *CAZ* Ceftazidime, *FEP* Cefepime, *AK* Amikacin, *LEV* Levofloxacin, *CIP* Ciprofloxacin, *CO* Colistin, *TZP* Piperacillin/tazobactam, *SCF* Cefoperazone/sulbactam, *ATM* Aztreonam, *CAV* Ceftazidime/avibactam, *GM* Gentamicin

^b^ *P* value was calculated through the resistance rates between ICU inpatients and healthy individuals

### Molecular characterization of *P. aeruginosa*

**Fig. 2:**
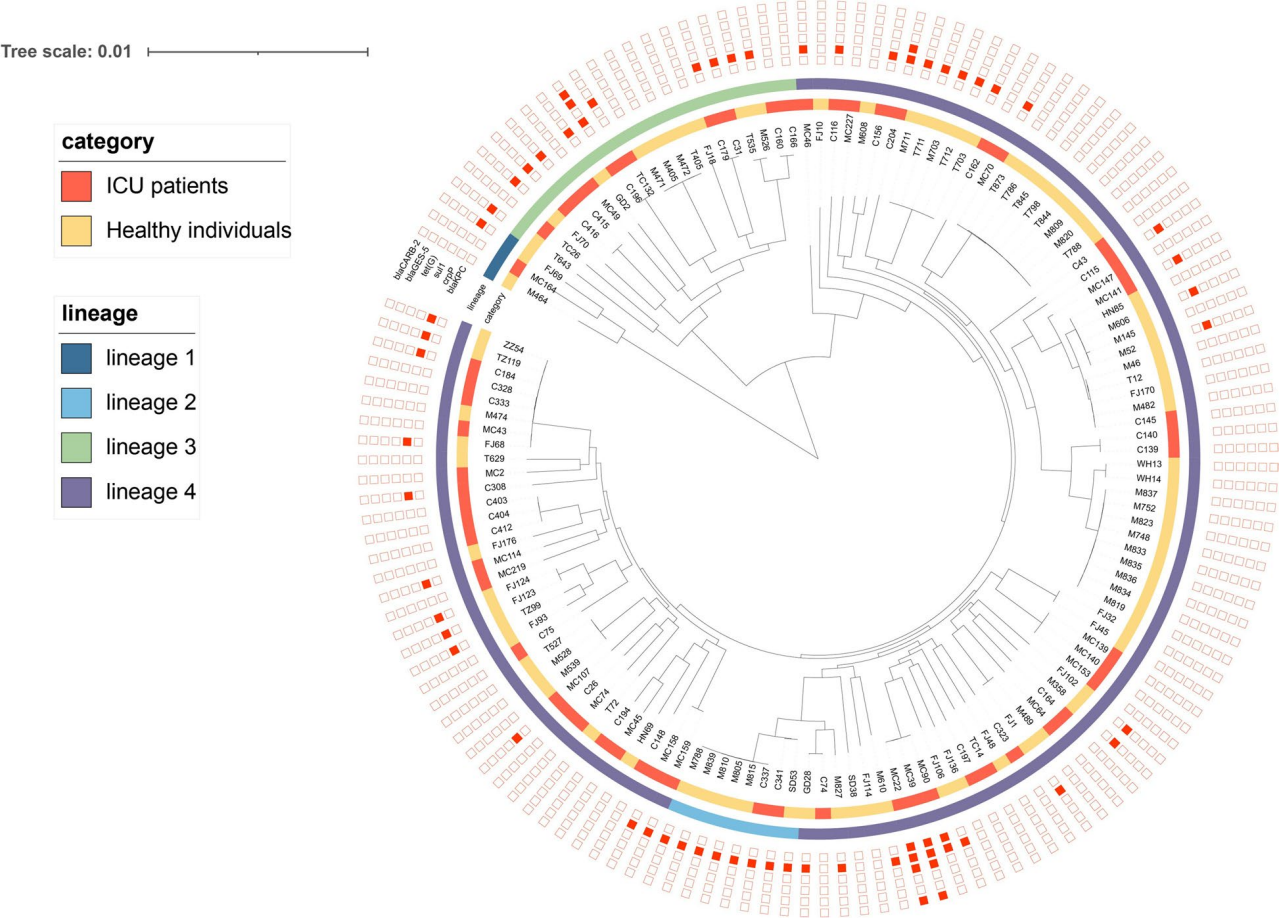
Phylogenetic tree of 140 intestinal *P. aeruginosa* strains, with their isolation category, lineages, and profiles of antimicrobial resistance genes. The rings consisting of small squares from the inside out indicated *bla*_KPC_, *crpP*, *sul 1*, *tet(G), bla*_GES-5,_ and *bla*_CARB-2_, respectively. The filled square represented the presence of a resistance gene

The core-genome-based phylogenetic analysis, together with BAPS was used to define lineages within the 140 sequenced intestinal *P. aeruginosa* strains. The BAPS analysis revealed four distinct lineages among the 140 isolates. The main lineage 4 (*n* = 109), comprised 46 intestinal *P. aeruginosa* isolates from ICU inpatients and 63 from healthy individuals (Fig. 2). The phylogenetic tree revealed diverse genetic backgrounds of *P. aeruginosa* strains from both ICU inpatients and healthy individuals (Fig. 2). Specifically, we observed 59 isolates from ICU inpatients were categorized into 41 distinct sequence types (STs) and 81 isolates from healthy individuals were categorized into 38 different STs. The most prevalent ST among isolates from healthy individuals was ST1405 (*n* = 9), followed by ST244 (*n* = 8) and ST381 (*n* = 8). However, The STs of *P. aeruginosa* from ICU inpatients were much more diverse than from healthy individuals. The most prevalent ST was ST274 (*n* = 4), followed by ST244 (*n* = 3), ST11, and ST463 (*n* = 3). Thirteen STs were found from the 19 CRPA strains obtained from ICU inpatients. Only two types, ST620 and ST1405, of the six CRPA strains from healthy individuals were identified, which were completely distinct from that of ICU-derived CRPA.

Five naturally-occurring and chromosome-borne genes (*aph3-IIb*, *catB7*, *bla*_PDC_, *fosA,* and *bla*_OXA-50_) were found in all *P. aeruginosa* isolates, according to sequence analysis. A total of 11 acquired resistance genes, including five for aminoglycoside resistance genes (*aac(6')-Ib4, aadA6, aphA15, aac(6')-IIa,* and *ant(2'')-Ia*), one for trimethoprim resistance (*sul 1*), one for tetracycline resistance (*tetG*), one for fluoroquinolone resistance (*crpP*), and three for β-lactam resistance (*bla*_CARB-2_ *,* *bla*_KPC_ and *bla*_GES-5_), were identified and varied greatly among the 59 ICU-derived isolates (Fig. 2). For instance, the *crpP* gene was identified in 40.68% (24 out of 59) isolates, three of which carried two identical copies. The other genes were each present in only a few isolates (*n* = 1 to 3). While in 81 isolates from healthy individuals, none of the other acquired resistance genes was observed except for the gene *crpP*. 39.51% (*n* = 32) and 1.23% (*n* = 1) process one copy and two copies of *crpP* gene, respectively.

We further determined the genetic environment of three KPC-producing *P. aeruginosa* isolates collected from ICU patients (two KPC-2 and one KPC-87, a KPC-2 variant) obtained in 2022 from three hospitals in the city of Hangzhou. KPC-87-producing strain MC22 showed deletion of the nucleotides GCA after position 721 in *bla*_KPC-87_, which results in the amino acid substitution of GT241A, and conferring ceftazidime/avibactam resistance. Genetic environment analysis showed that *bla*_KPC_ was flanked immediately by IS*Kpn6* in its downstream and IS*Kpn8* in its upstream, and TnpR and Tn3 further upstream, three *bla*_KPC_-containing isolates, therefore, showed the same conserved Tn*3*-TnpR-IS*Kpn8*-*bla*_KPC_-IS*Kpn6* genetic context.

Since the use of disinfectants has significantly grown both in hospitals and the environment since the emergence of 2019-nCOV, we also examined disinfectant genes based on whole-genome sequencing. The results revealed the presence of the antiseptic resistance gene *qac*E in five ICU-derived strains, three of which were identified as CRPA, and two co-carried the *bla*_KPC-2_ gene. Notably, two of the *qac*E-carrying strains belonged to the international high-risk clone ST235 with one CRPA, and two CRPA strains belonged to the potential high-risk clone ST463.

**Fig. 3:**
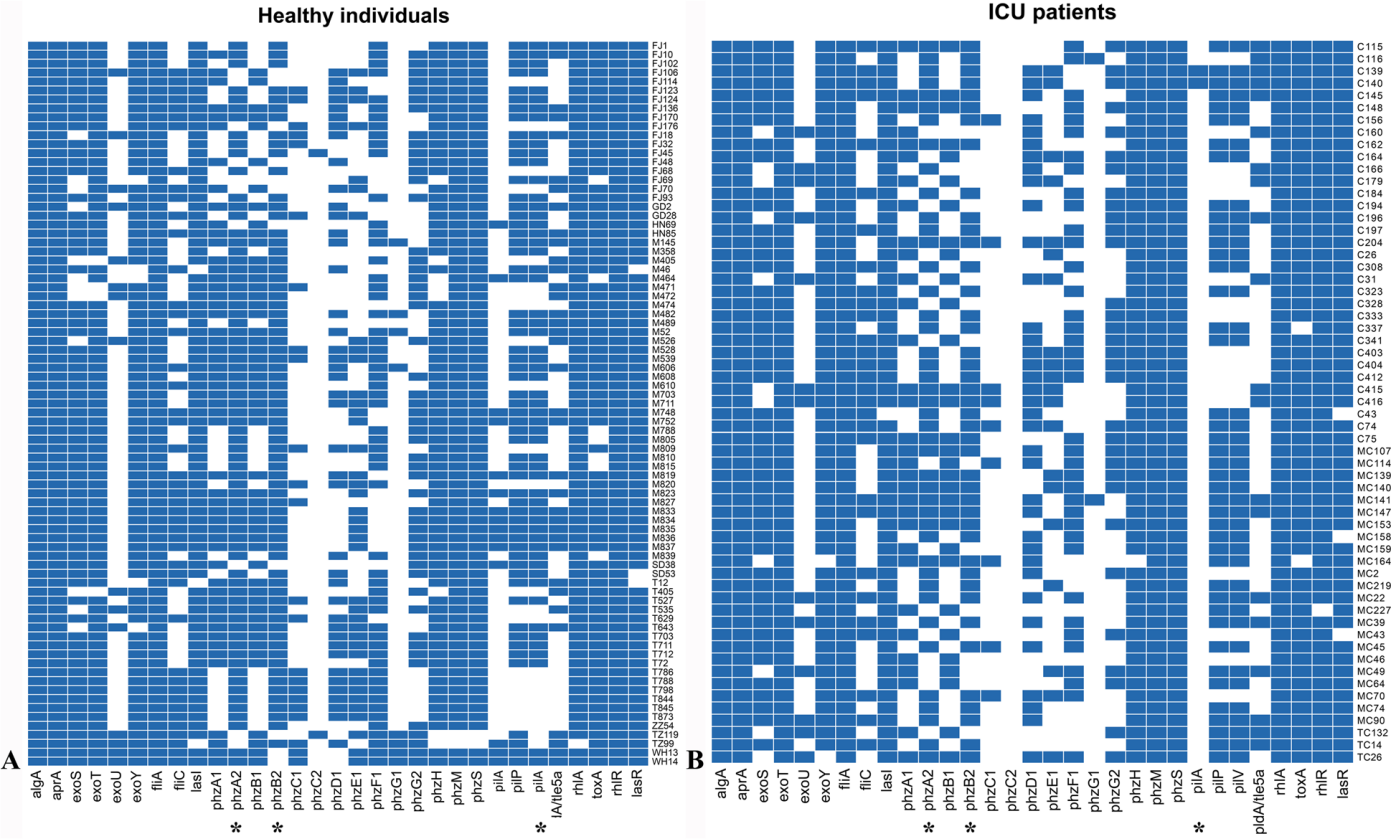
Virulence genes of 140 intestinal *P. aeruginosa* strains. **A** Strains collected from healthy individuals. **B** Strains obtained from ICU inpatients. Asterisk indicates a significant difference between the two groups

We also characterized the virulence in these isolates based on whole-genome sequencing analysis to identify the virulence genes of these intestinal *P. aeruginosa* isolates. Though the prevalence rates of these virulence genes varied significantly, only the prevalence of two phenazine biosynthetic genes (*phzA2* and *phzB2*) and one Type IV pili gene (*pilA*) were significantly different between the two groups (Fig. 3). The prevalence rate of *phzA2*, *phzB2,* and *pilA* in *P. aeruginosa* collected from ICU inpatients and healthy individuals were 72.88%, 71.19%, 3.39%, and 87.65%, 86.42%, 18.52%, respectively.

## Discussion

ICU stays were one of the most important risk factors for contracting CRPA [12]. Our findings also found that both the isolation rate of *P. aeruginosa* and the isolation rate of CRPA in *P. aeruginosa* isolates were substantially higher in the intestinal samples from ICU inpatients (10.28% and 32.20%) than that in healthy individuals (3.99% and 7.41%). To our knowledge, no literature has done a comparative study on the gastrointestinal *P. aeruginosa* carriage between healthy individuals and ICU inpatients. Interestingly, the carriage rate in this study (3.9%) is comparable to that in our prior work (4.0%), which involved intestinal samples from inpatients [13]. This is most likely because tigecycline in the agar plates was more selective and increased the isolation of *P. aeruginosa* for most other gram-negative bacteria are highly susceptible to tigecycline, while *P. aeruginosa* is naturally resistant to it. Furthermore, the MIC values of carbapenems in healthy individuals were lower than in ICU inpatients, as well as fluoroquinolones and β-lactam/β-lactam inhibitors. Combined with the molecular characteristics of the strains, we can speculate that ICU-derived *P. aeruginosa* has a higher risk of obtaining acquired resistant genes which resulted in higher MIC.

We can clearly see the disparities between these two groups using genomic analysis. First, ICU inpatients are more likely to acquire resistance genes, in addition to naturally carried chromosome-borne genes, only one acquired fluoroquinolone-resistance gene *crpP* was obtained in healthy individuals, on the contrary, *P. aeruginosa* isolates from ICU inpatients acquired 11 various resistance genes, such as tetracycline, aminoglycoside, and β-lactam resistance genes, which further explains the higher antibiotic MIC values in *P. aeruginosa* from ICU compared to the healthy individuals. In addition, no carbapenemase-encoding genes were found in CRPA strains gathered from healthy populations, pointing to the possibility of efflux, *bla*_PDC_ expression, or decreased permeability as potential resistance mechanisms. Fortunately, these resistance mechanisms do not contribute to horizontal transmission.

Alarmingly, we identified not only the *bla*_KPC-2_ gene, which was renowned for its high hydrolysis and transmission potential; but also the *bla*_KPC-87_ variant gene, which was first reported in *K. pneumoniae* [14], and it has not been reported in *P. aeruginosa* thus far. The amino acid deletion of GT241A in KPC-87 belonged to the loop between the β3- and β4-strands (Cys238 to Thr243; Loop238–243), one of the cores of the active site of KPC beta-lactamase [15]. *bla*_KPC-87_ gene can confer ceftazidime/avibactam resistance in *K. pneumoniae*, and the MIC value of the tested MC22 strain against ceftazidime/avibactam was 32 μg/ml, indicating this gene may also function well in *P. aeruginosa*. Though individual acquisition is more frequent than patient-to-patient transmission according to the MLST results, the genomic environments of three isolates that possess the *bla*_KPC_ gene were identical to our previously reported *P. aeruginosa* clinical isolates [16], suggesting the possibility of transmission of *bla*_KPC_ gene between various specimen types or microorganisms. We should pay close attention to this phenomenon as the finding of KPC-87 carbapenemase also indicates that ceftazidime/avibactam-resistant organisms may be increasing as a result of the use of this antimicrobial combination in clinical settings.

The use of disinfectants has probably increased because of the global 2019-nCOV outbreak. In contemporary hospitals, the issue of bacterial resistance to disinfectants is growing, which is comparable to the problem of antibiotic-resistant bacteria [17]. Previous studies have demonstrated that disinfectants facilitate the transformation of exogenous antibiotic-resistance genes [18]. In the current study, the five *qacE*-bearing strains were all recovered from ICU inpatients, where disinfectants are extensively applied, and three of them were identified as CRPA. These results indicated that frequent use of disinfectants might promote the widespread emergence of antibiotic resistance. Therefore, the ancient thoughts of the antibiotic resistance issue might be eased by the increased use of disinfectants might not be true at least in this case.

Except for the virulence genes *phzA2*, *phzB2*, and *pilA*, which were greater in healthy individuals, there was no noticeable difference between healthy individuals and ICU inpatients in the carriage rate of virulence factors. This may be because the accumulating resistance mutations in ICU-derived *P. aeruginosa* are linked to a decrease in the virulence [19]*.* However, it was interesting to find that our collection of three KPC-producing *P. aeruginosa* strains all belonged to the high-risk clone ST463. In the meantime, two of the five *P. aeruginosa* strains carrying *qacE* belonged to the high-risk clone ST463 that produces KPC, two of them belonged to the high-risk clone ST235, and one of them had the carbapenem resistance gene *bla*_GES-5_.

Although we did a systematic comparative study on CRPA prevalence, antimicrobial susceptibility, the prevalence of acquired drug resistance genes, and the presence of disinfectant resistance genes between intestinal *P. aeruginosa* isolates collected from healthy individuals and ICU inpatients. We acknowledge several limitations in this study. First, for the sampling, the provinces where fecal samples were collected inconsistently between the two groups, which can be biased. Second, we did not have data regarding medications before ICU admission, and samples were collected randomly during the hospital stay but not on hospital admission. However, despite these limitations, our study provides important information on prevalence of CRPA and molecular characterization of the two groups. We can conclude that the clinic should pay close attention to the environment of the ICU ward as well as the fecal samples of ICU inpatients. The intestine of ICU inpatients may represent an important reservoir of CRPA, possibly contributing to person-to-person transmission, and even transmission to the environment by these colonized persons.

## Conclusions

Higher prevalence, antibiotic resistance percentages, and acquired resistance genes of *P. aeruginosa* and CRPA were observed in ICU inpatients than in healthy individuals. The individual acquisition is more frequent than patient-to-patient transmission according to the polyclonal population structure. However, the identification of three KPC-producing multi-drug resistant strains reveals that a small-scale transmission may still occur and will pose greater challenges for clinical management.

## Materials and methods

### Isolates collection

This study was approved by the Ethics Committee of The Second Affiliated Hospital of Zhejiang University School of Medicine and consent was given by participants. All participants who accepted routine annual physical examinations were considered as healthy individuals and were included in this study and we collected fecal samples without selecting the test population for age or gender. Samples from ICU inpatients were collected during their hospital stay. Finally, a total of 2,605 non-duplicated fecal samples were collected from 2021 to 2022 from ICU inpatients in Zhejiang province and healthy individuals from ten provinces in China. For each sample, approximately five grams of each fresh fecal sample were suspended in 5 ml of LB broth and enriched overnight at 35 ºC without shaking. Then, 100 μl of the overnight enriched broth was inoculated onto a selective China-Blue agar plate containing 2 μg/ml tigecycline because *P. aeruginosa* is intrinsically resistant and incubated overnight at 35 ºC. Colonies on the plate were selected for further purification and confirmation as *P. aeruginosa* using matrix-assisted laser desorption ionization-time of flight mass spectrometry (MALDI-TOF MS) (Bruker Daltonik, Bremen, Germany). Only one suspected *P. aeruginosa* isolate was analyzed per sample.

### Antimicrobial susceptibility testing

The susceptibility of *P. aeruginosa* strains to commonly used antimicrobial agents was determined using the broth microdilution method in accordance with the Clinical and Laboratory Standards Institute (CLSI) guidelines. We analyzed 14 antimicrobial agents, namely imipenem (IPM), meropenem (MEM), ceftazidime (CAZ), cefepime (FEP), amikacin (AK), levofloxacin (LEV), ciprofloxacin (CIP), colistin (COL), piperacillin/tazobactam (TZP), cefoperazone/sulbactam (SCF), aztreonam (ATM), ceftazidime/avibactam (CAV), and gentamicin (GM). The minimum inhibitory concentrations (MICs) were interpreted according to the CLSI M100-S31 guideline for *P. aeruginosa* [20]. We considered strains to be CRPA if they showed resistance to imipenem or meropenem.

### Whole-genome sequencing and bioinformatics analysis

Genomic DNA was extracted using the PureLink Genomic DNA Mini Kit (Invitrogen, USA) following the manufacturer’s instructions. The DNA samples were sent to Novogene (Novogene, China) for whole genome sequencing on an Illumina NovaSeq PE150 platform. De novo assembly was performed using SPAdes version 3.15.1 [21]. Species identification and multilocus sequence types (MLST) were conducted using the PubMLST website. Acquired antibiotic resistance genes were analyzed by Kleborate version 2.1.0. Virulence factors and disinfectant resistance genes analysis was conducted using abricate. Advanced Heatmap Plots were performed using the OmicStudio tools at https://www.omicstudio.cn. A core-genome-based phylogenetic tree was generated using Parsnp in the Harvest package [22]. The lineages of the phylogenetic tree were defined using the Bayesian analysis of the population structure (BAPS) version 6.0 [23]. The molecular features of each strain were visualized using the online tool iTOL [24]. Comparison of genetic contexts carrying the *bla*_KPC-2_ was undertaken using the online NCBI basic local alignment search tool (BLAST) and visualized by Easyfig. Genome sequences were annotated by the RAST tool. For strains with missing sequences around *bla*_KPC_, a PCR mapping approach was adopted to compare the genetic context of the *bla*_KPC_ gene as described in our previous study [13].

### Statistical analysis

Statistical analysis was conducted by performing the Chi-squared test or Fisher’s exact test via the IBM SPSS software (version 23.0); *P* < 0.05 was considered statistically significant.

## Data Availability

The datasets used and/or analysed during the current study are available from the corresponding author on reasonable request.

## Abbreviations

ICU: Intensive care unit
CRPA: Carbapenem-resistant *P. aeruginosa*
MLST: Multilocus sequence type
MIC: Minimum inhibitory concentration
PCR: Polymerase chain reaction
CLSI: Clinical and Laboratory Standards Institute guideline
IPM: Imipenem
MEM: Meropenem
CAZ: Ceftazidime
FEP: Cefepime
AK: Amikacin
LEV: Levofloxacin
CIP: Ciprofloxacin
CO: Colistin
TZP: Piperacillin/tazobactam
SCF: Cefoperazone/sulbactam
ATM: Aztreonam
CAV: Ceftazidime/avibactam
GM: Gentamicin
CI: Confidence interval
